# Community Perspectives on Japanese Encephalitis Risk and Prevention in an Endemic Region of Australia

**DOI:** 10.1111/ajr.70227

**Published:** 2026-06-23

**Authors:** Jennifer White, Peter Murray, Megan Vilder, Sharon Saxby, David N. Durrheim

**Affiliations:** ^1^ Health Protection, Hunter New England Local Health District Wallsend New South Wales Australia; ^2^ School of Medicine and Public Health, University of Newcastle, University Drive Callaghan New South Wales Australia

**Keywords:** arbovirus, Australia, Culex, Japanese encephalitis virus, qualitative, vaccination, vector‐borne disease

## Abstract

**Objective:**

Japanese encephalitis (JE) is a mosquito‐borne disease caused by JE virus (JEV) infection, detected for the first time in south‐eastern Australia in 2022. In New South Wales (NSW), detections of JEV in mosquitoes and animal hosts, human JE cases, and climate and environmental considerations have informed which areas are considered high risk for JEV and which populations are eligible for vaccination (funded by Australian states and territories). However, early evidence indicates slower‐than‐expected uptake in these high‐risk areas. We aimed to explore how community members and healthcare professionals (HCPs), including general practitioners (GPs), pharmacists, and nurses, perceive and have responded to JEV risk through vaccination and personal mosquito‐bite prevention practices.

**Setting:**

Tamworth is classified as high‐risk for JEV by NSW Health.

**Participants:**

Semi‐structured interviews with community participants (*n* = 15), GPs (*n* = 7), nurses (*n* = 3), and pharmacists (*n* = 3).

**Design:**

An interpretative qualitative study. Data were analysed using an inductive thematic approach.

**Results:**

Three themes were identified: (1) Risk awareness shaped by experience, not policy: “I didn't realise Tamworth was identified as an area as well, I just was totally unaware,” (2) Vaccine eligibility does not translate into uptake: “There isn't really much promotion at the moment,” and (3) Building community‐level preparedness through communication: “Messaging that the whole community knows about.”

**Conclusion:**

Despite early public health efforts, awareness of JE and uptake of preventive measures remained limited in a high‐risk regional setting. Supporting trusted healthcare providers with clear, consistent communication is critical to optimising JE vaccine uptake.

## Abstract (highlights)

**What is already known on this subject:**

- Japanese encephalitis virus (JEV) emerged in south‐eastern Australia in 2022 and is now considered likely endemic in regional New South Wales, particularly west of the Great Dividing Range.
- JEV can cause severe neurological disease, and prevention relies on vaccination and personal mosquito‐bite protection.
- Although JEV vaccination is funded for eligible populations in high‐risk areas, early evidence suggests low uptake in regional communities.
- Rural vaccine uptake is influenced by access, workforce capacity, risk perception, and healthcare provider recommendation.

**What this paper adds:**

- This study provides qualitative insights into community and healthcare professional perceptions of JEV risk and prevention in a high‐risk regional NSW setting.
- Risk awareness was shaped more by personal experience than by official high‐risk designations or policy guidance
- Confusion about vaccine eligibility and funding—among both community members and some healthcare professionals—limited vaccination uptake.
- Practice nurses and community pharmacists played a key role in facilitating opportunistic vaccination and improving access for rural populations.
- Pulsed community‐wide communication is needed to support mosquito‐borne disease prevention in rural Australia.

## 1 Introduction

Mosquito‐borne viruses are a well‐recognised global health threat. Japanese encephalitis (JE) is a mosquito‐borne disease caused by the Japanese encephalitis virus (JEV) and is a leading cause of viral encephalitis across Asia and parts of the Western Pacific region [1]. Historically, JEV activity in Australia has been limited to sporadic cases detected in Queensland's Cape York Peninsula and the Torres Strait Islands [2, 3]. However, in 2022, JEV was detected for the first time in humans, pigs, and mosquitoes across multiple south‐eastern Australian states, including New South Wales (NSW), Victoria, and South Australia [4, 5, 6, 7]. Since 2022, JEV has continued to be detected in humans, mosquitoes, and animals, suggesting that JEV is now likely endemic in south‐eastern Australia [8, 9, 10].

Most JEV infections are asymptomatic and less than 1% of cases progress to severe encephalitis [1]. A recent serosurvey in Northern Victoria, including locations where JEV had previously been detected only in animals, demonstrated a substantial burden of non‐clinical infection among people encountering the virus for the first time [11]. Similarly, a serosurvey conducted in NSW identified evidence of prior JEV exposure among residents, suggesting that transmission may occur beyond recognised clinical cases [12]. Among symptomatic cases, mortality rates may reach 30%, and many survivors experience long‐term neurological disability [13, 14]. Particularly in affected regions, health communication plays a critical role by promoting personal protective practices (PPP) against mosquito bites, such as wearing loose‐fitting long‐sleeved clothing, using mosquito repellents, and avoiding periods of peak mosquito activity [15]. Previous research emphasises the importance of providing tailored, culturally relevant information to communities at risk to support the uptake of protective behaviours [16, 17]. Given the severity of disease, the potential for long‐term neurological disability among survivors, and the associated health‐system costs, prevention through JEV vaccination has been prioritised in specific high‐risk regions across multiple Australian states and territories. In NSW, vaccination is recommended for all individuals aged 2 months and over who live or work in designated high‐risk areas [18].

Despite these recommendations, previous research has identified significant barriers to the uptake of recommended vaccines, issues which are often amplified in rural communities due to limited access to vaccination services, workforce shortages, long travel distances, and competing agricultural and seasonal work demands that constrain health‐seeking behaviours [19, 20]. In addition, the COVID‐19 pandemic contributed to increased public scepticism, misinformation, and fatigue around vaccination messaging, particularly in regional and remote areas [21, 22], creating further challenges for the acceptance of newly introduced vaccines such as JEV.

Since the initial emergence of JEV in south‐eastern Australia in 2022, Tamworth and the New England area of NSW have been the focus of substantial public health activity. This has included media coverage and targeted clinical alerts from the Australian Department of Health Disability and Ageing [8, 23], NSW Health [24], the Royal Australian College of General Practitioners (RACGP) [25], Primary Health Networks [26], Department of Primary Industries [27] and Australian Veterinary Association [28]. In parallel, free JEV vaccination has been made progressively available to eligible local government areas (LGA) in the New England region to support vaccine delivery in primary care and community pharmacy settings [18]. Despite these efforts, vaccine uptake has remained low, and awareness of JEV risk and prevention appears to have declined over time. Recent reports indicate that only approximately 8% of the population in Tamworth has been vaccinated against JEV [29]. The ongoing detection of JEV in animal and mosquito populations underscores the need to move beyond emergency‐phase messaging and better understand how communities perceive risk, prevention, and vaccination in an endemic context.

This study responds to this gap by exploring community perceptions of JEV risk and prevention in a regional city with intense formal public health messaging and vaccine availability. Conducted in Tamworth LGA, NSW, this study explores how community members and healthcare professionals (HCPs), including General Practitioners (GP), pharmacists, and nurses, perceive and have responded to JEV risk through vaccination and personal mosquito‐bite prevention practices.

## 2 Methods

### 2.1 Context

Tamworth is a regional city in north‐eastern NSW, Australia, located approximately 400 km north of Sydney, with an estimated population of 43 874 residents [30]. The region's economy is primarily based on agriculture, livestock production, and food processing, alongside growing health, education, and service sectors. Tamworth experiences a warm temperate climate with distinct seasonal variation and periodic flooding, creating favourable conditions for mosquito breeding in the wet summer months [31].

### 2.2 Theoretical Framework

This study explored how community members and HCPs perceive and have responded to JEV through vaccination and PPP practices using an interpretative qualitative study design. An inductive thematic approach [32] grounded in interpretive analysis was deemed appropriate for gaining a deeper understanding of the participants' perceptions and responses to JEV vaccination and PPP. This approach focuses on the meaning and intentions people ascribe to their own actions and their interactions with others [32]. The qualitative study was informed by the Consolidated Criteria for Reporting Qualitative Research (COREQ) checklist [33]. Ethics approval was obtained from the University of Newcastle Human Research Ethics Committee (2025/ETH00723).

### 2.3 Participants and Recruitment

Recruitment was open to community‐residing adults, GPs, pharmacists, and practice nurses working in Tamworth, NSW. An invitation email containing study information was distributed through multiple health professional networks, including an email staff distribution list (sent from the General Manager Tamworth Hospital), The New England General Practice Research Network, Pharmaceutical Society of Australia (PSA), and the Hunter New England Population Health Unit. Reminder emails were sent over several weeks to encourage participation. A total of 15 community adults, 7 GPs, 3 pharmacists, and 3 nurses consented to be contacted by the primary author to schedule interviews. Written and/or recorded consent was required from all participants before their interview commenced. Participant recruitment took place between August and November 2025.

### 2.4 Interviews

Individual videoconference interviews (between 30 and 45 min) were conducted by an independent, experienced qualitative researcher (JW). Guided by an interview schedule (Table 1), questions explored knowledge, attitudes and behaviours towards mosquito bite PPP and JEV vaccination, including barriers and facilitators. The iterative process of cumulative and concurrent data collection and analysis, incorporating a process of constant comparison, allowed emergent themes to inform continuing data collection [34].

**TABLE 1:** Semi‐structured interview guide exploring perceptions of Japanese encephalitis and vaccination.

Topic	Core question	Community participants – prompts	Health professionals – prompts
Background & role	Can you please start by telling me a little about yourself?	Household composition; children; living context	Current role; years of experience; involvement in vaccination delivery; patient groups
General immunisation practice	—	—	How immunisation discussions are conducted; triggers for vaccine conversations; patient groups
Perceived risk of mosquito‐borne disease	How concerned are you about diseases spread by mosquitoes?	Awareness of mosquito‐borne diseases and JE; perceived likelihood and severity; information sources	Awareness of JE; concern for patients; perceived risk groups (e.g., outdoor workers, piggery staff)
Mosquito‐bite prevention	What have you heard about mosquito‐bite prevention during mosquito season?	Avoidance strategies; repellents; clothing; vaccination; NSW Health advice and uptake	Avoidance strategies for self and others; repellents; clothing; vaccination; NSW Health advice and uptake
Awareness of JEV vaccine	What have you heard about the JEV vaccine?	Local recommendations; eligibility; positive or negative messages heard	Confidence discussing JEV vaccines; employer support; how advice is given
Vaccine decision‐making	Have you received (or delivered) a JEV vaccine?	Reasons for accepting or declining; workplace availability; prior adult vaccination	Experience delivering JEV vaccines; how discussions were initiated; patient responses
Hesitancy & concerns	How do you respond when concerns are raised about vaccination?	Sources of reassurance; role of family, friends, community or religious leaders	Approaches to hesitancy; what helps or hinders vaccine acceptance
Access & logistics	Can you take me through how you would go about getting a JEV vaccine?	Location; time off work; transport; costs; trust in provider	Practical barriers to delivery; system or workflow issues
Closing	Is there anything else you'd like to add?	Open reflections	Open reflections

*Note:* Interviews were conducted by experienced qualitative researchers using a flexible, non‐verbatim approach.

### 2.5 Data Analysis

Interviews were audio recorded and transcribed verbatim by an external transcription service, with identifying data removed. Transcripts were checked for accuracy by the primary author (JW) and analysed using an inductive thematic approach [32]. Each professional and the community group was analysed separately using the same approach, followed by a comparative synthesis to examine convergences and divergences. This involved: (i) identifying units of meaning using a process of reading the transcripts line‐by‐line; (ii) grouping units into categories whereby each category was labelled to assist with retrieval between the data; and (iii) examining relationships between codes in the context of the research question to form themes. To ensure accuracy, consensus coding was undertaken by three researchers (JW, MV, SS). Any differences in researcher perspective were resolved by negotiation and, if necessary, codes were regrouped and recoded until consensus was reached. The resulting themes and sub‐themes were then shared with the full authorship team (spanning clinical and academic backgrounds) for critical dialogue and refinement. Trustworthiness of our data was upheld using approved strategies within qualitative research including purposive sampling (credibility), immersion in data (credibility), field notes (dependability), reflexive analysis (confirmability), and peer debriefing (confirmability and dependability) [35]. These strategies helped ensure that the researchers remained open to the data and did not demonstrate bias due to preconceptions inherent to their clinical status and experience [35]. Member checking was not undertaken to reduce the time burden on busy rural clinicians, where workforce shortages exist.

### 2.6 Results

A total of 15 community adults, 7 GPs, 3 pharmacists and 3 nurses from Tamworth were interviewed. Provision of demographic data is limited to protect participant anonymity. Findings were integrated and we identified three themes:

1. Risk awareness shaped by experience, not policy: ‘I didn't realise Tamworth was identified as an area as well, I just was totally unaware.’
2. Vaccine eligibility does not translate into uptake: ‘There isn't really much promotion at the moment.’
3. Building community‐level preparedness through communication: ‘Messaging that the whole community knows about.’

Exemplar quotations are provided to evidence each theme. Participants are noted as ‘C’ for community adults, ‘GP’ for GP participants, ‘P’ for pharmacy participants and ‘N’ for nurse participants.

## 3 Results

### 3.1 Theme 1. Risk Awareness Shaped by Experience, Not Policy: ‘I Didn't realise Tamworth was Identified as an Area as Well, I Just was Totally Wnaware’

#### 3.1.1 JEV Awareness

Across both community participants and HCPs, awareness of JEV was variable. Many community participants reported first learning about JEV through study recruitment materials or isolated media messages. Most expressed surprise that Tamworth had been identified as a high‐risk area and noted a lack of recent, visible information in their community (e.g., posters, news coverage, social media).

I only know about it because of your study… Haven't seen a single poster or ad [advertisement] anywhere. (C1)

Awareness among HCPs was also inconsistent and participants attributed this to sporadic public messaging and limited workplace communication. Early media coverage was described as brief and unsustained, leading to reduced visibility and confusion about risk and vaccine eligibility.

I know it was on the radio and the newspaper and the TV when it first came out, just brief snippets. I have not really heard it anywhere else. (P2)

I haven't read much about it recently. I think it's transmitted through mosquito bites or something like that. (GP4)

While most HCPs perceived that there was health risks associated with JEV, many framed their knowledge as limited and historically linked to travel medicine. Several GPs noted that low clinical exposure and few patient‐initiated requests for vaccination meant JE was not routinely ‘front of mind’ (GP5).

Most participants acknowledged that their own media habits limited exposure to formal health messaging. Many described not watching the television or buying newspapers and instead relied on local radio, social media, and both formal and informal networks for information.

I do not watch the news. (GP1)

Pretty much, in the rural setting, it is word of mouth and experience. So, if someone has a friend that has had it, it is pretty much like a bush telegraph. (C15)

#### 3.1.2 Perceived Personal Risk

Perceptions of risk varied with some participants reporting being vulnerable, particularly those with existing health issues or who felt they were prone to mosquito bites, while others perceived their risk as low and were less concerned. Parents reported heightened concern for their children.

I spend a lot of time inside… the likelihood of severe symptoms seemed quite low… it's not at the forefront of my mind. (P1)

I am particularly aware of the youngest one, the mosquitoes just love him‐I think that he is almost allergic to them, because he just scratches uncontrollably, and they just get horrible. (C14)

Community participants typically linked the risk of mosquito bites with environmental cues. Living on properties near water or, falsely, near swimming pools, or engaging in outdoor recreation (e.g., camping, hunting) was reported to increase exposure. However, when participants heard about local cases they reported increased concern.

Well, because I have got a swimming pool, I have got mosquitoes at my house.(C11)

I think [I am more concerned] since I know of someone …. I am much more worried. I look at him [person with JEV] and I think, ‘Wow, he was quite healthy and well’—not an age category that I would think would be susceptible to it necessarily—or to become so unwell. (C8)

#### 3.1.3 Personal Protection Practices

All participants reported using a range of mosquito‐avoidance strategies, including repellents, long clothing, mosquito coils, and thermacells. Some shifted outdoor activities to earlier in the day or moved activities indoors at dusk. However, concerns about chemical exposure, especially for children, limited the consistent use of repellents containing DEET (N, N‐Diethyl‐meta‐toluamide).

I feel funny about spraying the Aerogard (insect repellent). I happily do it to myself, but I feel like it is quite toxic. I do not love spraying it on them [children] all the time. So, I guess we probably tend to move inside once, you know, dusk and the mozzies start coming out—it probably forces us to go inside, which is a bit of a shame. (C8)

Several hobbyist pig hunters reported having heightened awareness of mosquito borne viruses but reported inconsistencies with PPP use.

By nature of the sport, we wear jeans and boots and long sleeves and that's working.’ (C5)

Likewise, while hunting was described as a family activity participants indicated that were not likely to use repellents on children, due to their own discomfort

I don't want to put that on the kids, it's gross and, you know. (C4)

Travel experiences often shaped awareness and PPP. Participants who had travelled to JEV‐endemic regions recalled receiving information or vaccination prior to their overseas trip and described more rigorous protective practices when overseas than at home.

Definitely [I use a repellent] anything with DEET in it. And I'm much more on the repellent when I'm overseas. (C7)

### 3.2 Theme 2: Vaccine Eligibility Does Not Translate Into Uptake: “There Isn't Really Much Promotion At the Moment”

#### 3.2.1 Confusion and Vaccination Barriers

Most community participants reported having not previously heard about the JEV vaccine. Only a small number of all participants had been vaccinated, typically due to their predominantly outdoor lifestyle, work or travel‐related risk. Several reported that participation in the study prompted them to consider vaccination; however, some encountered uncertainty among GPs regarding eligibility.

I felt she [GP] put it off though… like they are only offering it to people that are immunocompromised. (C1)

I was speaking to my doctor, and she … as far as she was aware, you actually had to be a worker working in the agricultural or directly with the pigs. And she thought otherwise you had to pay for it. (C3)

Few HCPs reported having prioritised JEV vaccination for themselves, and uncertainty regarding eligibility was evident. Some HCPs incorrectly believed that the JEV vaccine was included under the Australian Government's National Immunisation Program, which provides free vaccines nationally for selected diseases, whereas JEV vaccination is funded through state and territory programs for defined high‐risk groups. This uncertainty contributed to hesitancy and delayed uptake, even among those aware of local risk. As one pharmacist noted, “I take measures to prevent myself getting bitten… but I haven't gone and got the vaccine… there's a bit of a grey area… on who's allowed the free vaccine still” (P3). Similarly, a GP reflected, “The fact that it is now under the National Immunisation Program, is the main reason nobody was doing it before” (GP1), highlighting persistent confusion regarding vaccine funding and eligibility pathways.

Most community participants reported that their primary source of vaccination information was informal discussion with family, friends, and social networks. These conversations strongly influenced decision‐making and were reported as influential in rural communities, where information was said to spread quickly through trusted networks. However, community participants reported the impact of cost, GP appointment availability, and time constraints on vaccination uptake.

I think wait times out here is really hard. Like the last time I had to book in they said, oh if you want to see a regular GP, like the next time you will get to see them is in six weeks…So probably unless you have got, you know a special need to see your GP, it is not going to be on your radar to just pop in for a vaccination. (C12)

I mean in Tamworth getting into a GP is quite hard, even when you've got your regular GP, it is very difficult to get into a GP. (C15)

In the absence of formal vaccination campaigns, some HCPs promoted word‐of‐mouth conversation to try and increase the dissemination of JE information and reach high‐risk groups.

I believe, getting the word out there, because when I give them the vaccines, I tell them to mention to your friends you've had it, mention to other farmers that you've had it, mention to other energy workers that you've had it, you know what I mean. (P3)

#### 3.2.2 Competing Priorities

Pharmacists reported limited promotion of JE vaccination, with attention largely focused on other high‐profile seasonal vaccines.

I think everyone's sort of more worried about those other nasty ones at the moment. But yeah, as far as promotion in store, there isn't really much at the moment, but we're, we're looking at changing that. (P1)

We haven't done a great job of promoting, like any of the services, to be fair. (P1)

Pharmacists reported additional barriers to the promotion of JE vaccine including the need for training, inconsistent public promotion, and workforce shortages.

The first barrier was probably just the training… it's a free module… but for pretty much every other vaccine we don't need to. (P1)

We've started getting some more boots on the ground, so we're looking at really ramping up, especially our vaccination clinic. So, we're looking at improving that awareness amongst our patients at the moment, you know, just simple advertising and whatnot. (P3)

While GPs emphasised the value of prevention, limited consultation time and the complexity of patient needs often meant that vaccination promotion was secondary to addressing acute and chronic disease management.

So, we are really struggling and this kind of preventative maintenance stuff we try to do, but literally every single patient has a problem list of at least five and they are more serious problems…I think on a case to case basis…Patients have agendas… they don't really want health promotion areas they're not concerned about. (GP1)

Workflows where nurses reviewed patients before GP consultations (such as for other immunisations, health assessments, chronic disease management plans etc.) reportedly facilitated identification of the need for vaccines.

Yeah, for sure, you try and fit everything into, like, your 15 min, half hour, as much as you can. I like to sort of research the person before they come in and make notes saying, you know, due for Shingrix, JE all that sort of stuff—so, you know so it prompts me not to forget. (N1)

#### 3.2.3 Nurse‐Led Advocacy as Catalysts for Vaccination Uptake

Increased awareness and promotion of JE vaccination was attributed to funded vaccination and the leadership of practice nurses with immunisation expertise.

We have had it in stock since we knew that we were a hotspot, and we have offered it to all our patients that were eligible for it. (N1)

In several instances, nurses were described as champions for driving JE vaccination, identifying eligible patients, initiating vaccine‐related conversations, and supporting GPs.

I would have thought our vaccination rate here is quite reasonable simply because the way our practice works is that our nurses see virtually everyone beforehand, they are all nurse immunisers. (GP5)

Participation in the study prompted some GPs and nurses to consider system‐level changes, including electronic prompts and whole‐of‐practice approaches to place JE at the forefront of staff minds.

I will go to Best Practice and see if I can put in an action or reminder… use a systems approach… whole of practice. (GP2)

#### 3.2.4 Opportunistic Vaccination and Targeting Hard‐To‐Reach Populations

Across general practice and pharmacies, preventive activities were frequently described as occurring opportunistically. Most pharmacists offered online booking but highlighted the importance of walk‐in or same‐day appointments to accommodate the unpredictable schedules of agricultural workers and others less likely to plan.

Farmers feel like, oh, I could just do it today… they're that type of character… it suits them to be able to have a conversation about it and then go, could I get it today? And I say, yeah, no worries. (P3)

HCPs reported that opportunistic contact was particularly important for people, especially farmers and their families, who often saw the GP infrequently. HCPs were mindful that these encounters created opportunities for a broad review of preventive needs, including wellbeing and vaccination, including JEV.

Oh, look, how's your mental health going? How's the farm going… lists of medication… hospital discharges… it was something we would just pull up whenever we saw a patient who we knew just didn't come into town very much. (GP5)

So, if it was someone who once a year would have just a little bit of a checkpoint, kind of checklist thing that we would go through informally, yeah, and the vaccination became part of that. (GP6)

Some GPs described leveraging existing Q fever vaccination clinics conducted for at risk agricultural and abattoir workers to also offer JE vaccination since their presence was required “to do the skin test and order the blood forms.” (GP5)

We did a lot of Q fever vaccination clinics, [and] we would include Japanese encephalitis in that. Most people who came in for the Q fever would also get the JE vaccine. (GP6)

### 3.3 Theme 3: Building Community‐Level Preparedness Through Communication: “Messaging That the Whole Community Knows About”

#### 3.3.1 Need for Clear Guidance, Resourcing, and Promotional Infrastructure

Participants reported the need for consistent messaging and clearer eligibility guidance. HCPs noted the benefit of electronic clinical prompts and system supports to support JE vaccination. General practices reported opportunities for greater dissemination of information through practice Facebook pages, waiting‐room screens, and dissemination of the JE fact sheet (NSW Health).

So, the practice has a Facebook page that the patients can follow. We do have a big screen in the waiting room that they put promotions and things through so that's certainly another way. They're probably the two main ways that they would push things out to the patients, I don't think they generally kind of do bulk texts or emails or that sort of stuff. (N2)

Pharmacies should, like if they are in a hot spot for it, they should be saying, hey, have you thought about getting this done, you're out in the bush, if you're hiking and camping … you guys really should be looking at getting this done. (C6)

All participants advocated for promotion through community organisations. The need for messaging to ‘Be present in work and public functions’ (C5) such as rural shows, local festivals, and through key organisations was considered important for outreach.

I was just thinking like targeting big businesses or big employers lots of people might spread the word. (C8)

Although social media was seen as the most common information source, participants noted the need for paid or sponsored content to cut through competing messages.

I think that even on social media, like you're competing against so much, there's so much on there, it's so hard to get your point across, it's got to be stop scrolling worthy, you know. So, to get people to actually stop doing their little scroll, and go, hang on, what's this about? You know, you need something really eye‐catching to actually get people to listen these days. (5)

Participants emphasised the importance of respectful, age‐appropriate health messaging that was concise, engaging, and not patronising. One participant noted that creative, humorous campaigns, such as those from Queensland Health, as being effective for attracting attention while conveying serious messages. Participants referred to highly visible public health campaigns, such as Slip‐Slop‐Slap and breast cancer screening campaigns, as examples of communication approaches that successfully achieved broad community reach and message recall. Participants emphasised the importance of memorable, engaging, and repeated messaging to increase awareness of JE and encourage preventive action.

They [Queensland Health] get engagement because they're hilarious and a little bit left of centre… but they always have a message underlined and follow it up with health advice. (C7).

## 4 Discussion

This study explored community and HCPs' perceptions of JEV risk, prevention, and vaccination in a regional Australian setting classified as high risk for JEV. In Tamworth and the New England area of NSW, following the initial detection of JEV in 2022, there has been widespread media coverage, clinical alerts, and targeted professional communication. In subsequent years, eligibility criteria were clarified, and free vaccination was made available to populations living and working in designated high‐risk areas [18]. Our findings suggest that, over time, the visibility of JEV messaging has diminished, even though it is now likely endemic in these high‐risk areas.

A key finding in this study was that participants reported limited recent exposure to information about JEV and had demonstrated low awareness of disease severity, including the risk of long‐term neurological impairment, as well as limited understanding of vaccine availability. This finding is consistent with evidence that public health messaging often wanes once a disease becomes familiar, despite ongoing transmission risk [36]. Our findings support this contention and suggest that, in the absence of pulsed and refreshed communication strategies, both community awareness and professional attentiveness to JEV may diminish over time.

Mosquito‐borne viruses are an ongoing public health concern in Australia, and in addition to JEV include Ross River virus, Barmah virus, Murray Valley encephalitis virus and Kunjin (West Nile) virus. These infections can be difficult to detect due to non‐specific or asymptomatic clinical presentations [37]. To our knowledge, there have been relatively few studies examining community awareness of mosquito‐borne viruses and preventive behaviours in Australia over the past decade, with existing evidence consistently indicating low awareness of transmission pathways, symptoms, and bite avoidance strategies [38, 39, 40]. This study identified variation in knowledge of JEV risk in both community and HCP participants, which we posit may reflect participants' broader awareness of other mosquito‐borne viruses. In contrast, awareness of mosquito‐bite risk appeared linked to situational cues, such as flooding, increased mosquito activity, reported local cases, and overseas travel—reflecting the influence of established public health messaging. However, in the absence of pulsed and refreshed communication strategies, JEV risk was rarely considered, despite participants living in a high‐risk area; this is likely reflective of risk perception towards other mosquito‐borne viruses. Findings also indicated that PPP, including the use of repellents and avoidance of peak mosquito hours, was inconsistently applied. Concerns about chemical exposure, particularly for children, had the potential to constrain routine repellent use. These findings underscore the need to reinforce the safety of preventive behaviours that are consistent with an endemic mosquito‐borne threat.

Awareness of the JEV vaccine was limited among community participants, and confusion regarding eligibility was evident among both community members and some HCPs. Several participants reported receiving conflicting advice from clinicians, including assumptions that vaccination was restricted to specific occupational groups or required out‐of‐pocket payment. Notably, few HCPs reported prioritising vaccination for themselves, which may reflect both uncertainty about eligibility and low perceived personal risk. Given the well‐established role of trusted healthcare recommendations in shaping vaccine acceptance [41, 42], such uncertainty at the provider level may inadvertently reinforce low risk perception and reduce opportunities for opportunistic vaccination.

Despite these challenges, the findings highlight the central role of practice nurses and community pharmacists as key enablers of JE prevention. Nurse immunisers were frequently described as the primary drivers of vaccine identification and promotion within general practice. Pharmacists were valued for facilitating accessibility through cheaper, walk‐in vaccination options, particularly for agricultural workers and others with limited engagement with general practice. These findings align with the principles articulated in the NSW Health Immunisation Strategy [43], which emphasises workforce enablement, expanding vaccination settings, and reducing structural barriers to access as critical mechanisms for improving uptake. While opportunistic preventive actions were seen as beneficial, participants also emphasised the need for coordinated, staged communication strategies that reflect endemic risk. Regular, visible, community‐wide messaging delivered through trusted local channels—including healthcare settings, workplaces, community events, and social media—was viewed as essential to sustaining awareness and reinforcing the relevance of JE prevention. A pulsed approach could include early‐season messaging linked to rising mosquito numbers, escalation following detections in animal or mosquito populations, and intensified outreach if human cases are identified. Such an approach would support both personal protective practices (PPP) and vaccination uptake while reinforcing JE as an ongoing rather than episodic risk. Consistent with this, anecdotal observations from late 2025 suggest that physical in‐person outreach by Health Protection staff (Hunter New England Local Health District) to primary care services increased clinician awareness and engagement. This reinforces the value of direct, relationship‐based communication to complement broader public messaging, particularly when introducing or sustaining vaccination programs for emerging or endemic arboviral threats.

### 4.1 Strengths and Limitations

This study brings together perspectives from community members and multiple HCP groups within the same regional setting, enabling triangulation across stakeholder groups and providing a nuanced understanding of JE prevention in practice. However, findings should be interpreted in the light of several limitations. Participants were recruited from a single regional area, which may limit transferability to other JE‐affected regions with different demographics, risk profiles, or health service structures. Community participants were self‐selected and may have been more health‐engaged than the broader population, potentially underestimating gaps in awareness. Similarly, participating HCPs may have had greater interest in vaccination and public health than non‐participants. In addition, the study focused on community members and healthcare providers and did not include other key One Health stakeholders, such as animal health professionals, agricultural industry representatives, public health practitioners involved in surveillance and response, or policymakers. As a cross‐sectional study conducted relatively early in Australia's JE response, the findings may not capture evolving awareness or future changes in policy and practice. Future research incorporating a broader range of One Health stakeholders and examining experiences across diverse endemic and at‐risk regions would provide a more comprehensive understanding of JE preparedness, prevention, and control.

## 5 Conclusion

Awareness of JEV and its prevention remained fragmented in a regional Australian city classified as high risk for JEV, despite sustained traditional public health messaging to the community and HCP following its emergence. Since JEV is now considered an endemic threat, maintaining risk salience and seasonal preventive behaviours presents an ongoing challenge. Strengthening preparedness requires coordinated, visible communication strategies that integrate vaccination and mosquito‐bite prevention, supported by clear, timely guidance for trusted frontline healthcare providers. These findings have relevance not only for JE but also for future endemic and emerging arboviral threats in Australia.

## Author Contributions

**Jennifer White:** conceptualization, investigation, writing – original draft, methodology, writing – review and editing, formal analysis, data curation, supervision, project administration, validation. **David N. Durrheim:** conceptualization, funding acquisition, writing – original draft, methodology, writing – review and editing, formal analysis, supervision, validation, resources. **Sharon Saxby:** writing – review and editing, formal analysis, validation. **Peter Murray:** conceptualization, writing – original draft, methodology, writing – review and editing, formal analysis, validation. **Megan Vilder:** formal analysis, writing – review and editing, validation.

## Funding

The study was conducted with funding from Hunter New England Local Health District, and JW was funded by New South Wales Health through the Prevention Research Support Fellowship.

## Ethics Statement

Approval for this project was obtained from the Hunter New England Local Health District Human Research Ethics Committee (2025/ETH00723).

## Consent

The authors have nothing to report. All participants provided written informed consent.

## Conflicts of Interest

The authors declare no conflicts of interest.

## Data Availability

The data that support the findings of this study are available from the corresponding author upon reasonable request.
